# Rapid terahertz beam profiling and antenna characterization with phase-shifting holography

**DOI:** 10.1038/s41598-024-71641-7

**Published:** 2024-09-10

**Authors:** Michal Mrnka, Harry Penketh, David B. Phillips, Euan Hendry

**Affiliations:** Department of Physics and Astronomy, University of Exeter, Exeter, EX4 4QL UK

**Keywords:** Electrical and electronic engineering, Terahertz optics, Imaging techniques

## Abstract

In this paper we investigate the application of phase-shifting digital holography for the real-time characterization of electromagnetic sources in the THz frequency range. We use an off-the-shelf terahertz detector array composed of $64 \times 64$ power-sensitive pixels, over an area of $96\,\hbox {mm} \times 96\,\hbox {mm}$, to record intensity interferograms cast between the coherent radiation emitted from a reference source and an unknown antenna under test. This approach parallelizes the acquisition process with respect to conventional near-field point scanning methods, reducing the measurement time by orders of magnitude. In our system, the measurement time is limited only by the refresh rate of the detector array and the speed of a delay line stage that is used to phase-shift the reference wave. As a proof-of-principle demonstration, we map the 2D near-field distribution and estimate the far-field radiation pattern emitted by a plano-convex PTFE spherical lens antenna illuminated by a diagonal horn at 290 GHz frequency with $\sim \,1\,\hbox {Hz}$ refresh rate.

## Introduction

Holographic techniques have been widely adopted in the microwave and terahertz bands, taking inspiration from their optical counterparts^1–4^. Holography offers an alternative to (super)heterodyne mixing based techniques in applications like beam profiling^5–7^ and antenna measurements, where high frequency electromagnetic fields are to be characterized. In particular, holographic techniques can deliver cost and time effective solutions when testing electrically large structures^8–10^, since conventional methods such as near-field scanning and compact antenna test range measurements^11^ can be expensive, time consuming and/or practically unrealizable.

**Fig. 1:**
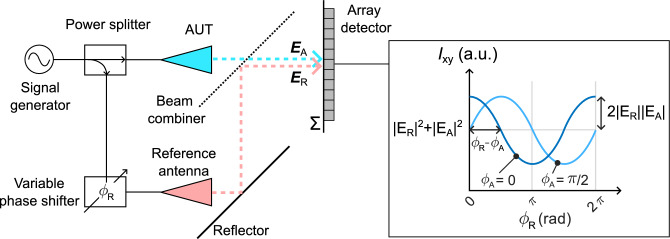
Principle of phase-shifting holography. The unknown electric field distribution $E_{{\text{A}}}(x,y)$ in the plane of the detector array $\Sigma$ is determined from an interference with a known reference field $E_{{\text{R}}}(x,y)$ on a pixel-by-pixel basis from measured intensity *I*(*x*, *y*).

Digital holography enables the phase structure of optical fields to be measured with amplitude-only (or optical intensity *I* only) detectors in what is sometimes referred to as a “phase-less” approach^12,13^. The phase information of the unknown field $E_{{\text{A}}}$ can be inferred from the intensity pattern cast by the interference of the unknown field with a known reference field $E_{{\text{R}}}$ (see Fig. 1). Modern holographic approaches mainly fall into one of two groups: off-axis holography^12,14–17^ and phase-shifting holography^18–21^. Both of these techniques are normally implemented at microwave frequencies with a single detecting probe scanning over a plane, which despite its accuracy can be a highly time consuming process, especially for electrically large antennas. Off-axis techniques usually rely on a free-space interference pattern between the unknown field and an obliquely incident reference plane wave. By analyzing and filtering the signal in Fourier space, the complex information is revealed from a single scan with a reduced effective pixel density given by the Fourier filtering process. On the other hand, the phase-shifting technique operates on pixel-by-pixel basis and preserves the spatial resolution of the acquired data. Multiple measurements for each position on the measurement plane are required, where the phase of the reference wave $\phi _{{\text{R}}}$ is varied (see Fig. 1).

At microwave frequencies, the interference in the phase-shifting approach has been applied through circuit based solutions (so called synthetic reference). The fields from the detector couple to a transmission line mode and a circuit based combiner is used to combine the unknown signal with a reference line. This approach is thus inherently lossy and expensive at THz frequencies. The free-space distribution of the reference signal in THz bands was proposed in theoretical paper^22^ and has very recently been experimentally implemented^23^ to characterize a point spread function of cryogenic instrument in a large sub-mm-wave radiotelescope. A comparison of the off-axis and phase-shifting approaches for mm-wave imaging with a detector identical to the one in this study has recently been published in Ref.^24^.

In phase-shifting holography (Fig. 1), the measured intensity *I* is given for each pixel at position (*x*, *y*) as:

1 $$\begin{aligned} I(x,y, \phi _R) = |E_R(x,y)|^2+|E_{A}(x,y)|^2 + 2|E_R(x,y)||E_{A}(x,y)|\cos {[\phi _{R}(x,y)-\phi _{A}(x,y)]}, \end{aligned}$$

where $E_{{\text{R}}}$ and $\phi _{{\text{R}}}$ correspond to amplitude and phase of the reference field. $E_{{\text{A}}}$ and $\phi _{{\text{A}}}$ represent the unknown amplitude and phase of the measured beam and $\phi _{{\text{R}}}$ is stepped through at least 3 values to recover the phase $\phi _{{\text{A}}}$ unambiguously^25^. Equation (1) assumes co-linearity between the reference and unknown fields and the polarization orientation of the linearly polarized array elements. To fully describe the polarization state over the plane, an orthogonally polarized reference field is also required. Co- and cross-polarization components of the field in suitable reference frame could thus be evaluated.

In this paper, we propose a real-time measurement technique based on the phase-shifting digital holography in combination with a commercial, room-temperature THz detector array to characterize the complex fields of propagating beams. The technique represents a fast alternative to time-consuming probe scanning approach for scans over areas up to 96 $\times$ 96 $\hbox {mm}^2$. Upon near-field to far-field transformation the far-field radiation pattern of an electrically large antenna is predicted at 290 GHz.

**Fig. 2:**
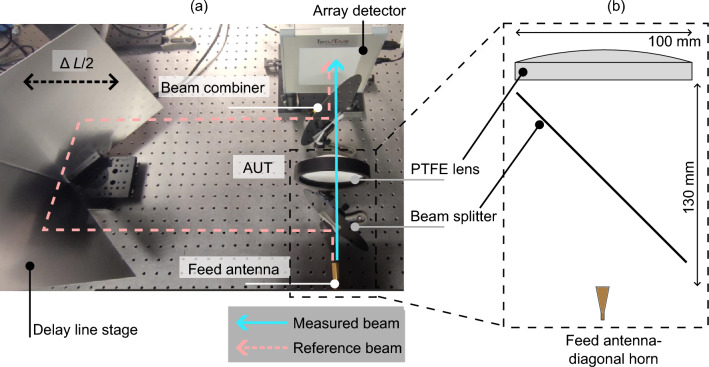
(**a**) Experimental setup. The measured beam is formed by a diagonal horn (bottom right corner) illuminating a spherical PTFE lens. The reference field is derived from the diagonal horn by a beam splitter and the path length of the reference beam is varied using the delay line stage. (**b**) Detail of the AUT formed by a diagonal horn and a PTFE lens—the beamsplitter also becomes part of the AUT in our setup.

## Results

### Complex beam profiling

The experimental setup for the phase-shifting holography employed in this paper is outlined in Fig. 2a. The beam under investigation (i.e. antenna under test—AUT) is formed of a plano-convex, spherical PTFE lens^26^ illuminated by a linearly-polarized diagonal horn antenna with − 3 dB beamwidths of 13$^\circ$ and 12 $^\circ$ in H- and E-plane, respectively^27^. The focal length of the lens is 151 mm and the diameter $D = 100\,\hbox {mm}$. The aperture of the horn is placed at a distance 130 mm from the lens (which is the back focal length of the lens) and the plane of the detector array is separated by distance 151 mm from the lens (see Fig. 2b for a photograph of the AUT setup).

The AUT is thus placed at a distance $d = 151\,\hbox {mm}$ from the detector array. Where coherence length is a concern, the reference signal can be generated from the same RF source that feeds the lens antenna via a directional coupler. In our measurement setup we apply quasi-optical beam-splitting in free space instead of the directional coupler. Alternatively the two signals can be generated by two independent sources tuned to identical frequency. The radiation from the AUT and the reference path are made to interfere in the plane of the detector via a beam combiner placed at $45^\circ$ with respect to the optical axis connecting the AUT and the center of the detector array. We use $675\,\upmu \hbox {m}$-thick high resistivity silicon wafers with 6-inch diameter for the beam splitting and combination.

**Fig. 3:**
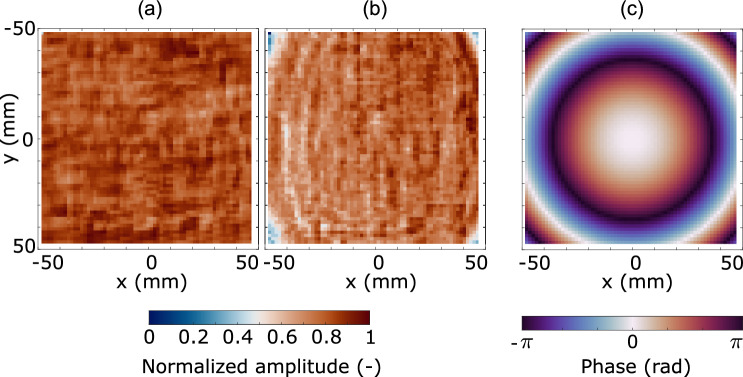
Imaging artefacts and reference field. (**a**) Spatial sensitivity of the array pixels—i.e. measurement of a uniform illumination. (**b**) Measured amplitude of the reference field $|E_{{\text{R}}}(x,y)|$ in the detection plane $\Sigma$—see Fig. 1. Dark corners are caused by the limited area of the circular beam combiner. (**c**) Spatial distribution of the reference field phase $\phi _{{\text{R}}}(x,y)$ determined by a simulation under spherical illumination assumption and distance given by Fig. 2.

To record the intensity *I*(*x*, *y*) of the interfering fields, a commercial detector array from TeraSense is used (Tera-4096 $64 \times 64$ pixel arrayed GaAs high mobility heterostructure). The pixels are linearly polarized and tuned to 290 GHz, the detection area is $96 \times 96\,\hbox {mm}^2$ with pixel pitch 1.5 mm. The pixels have sensitivity of $\hbox {NEP} = 1\,{{\text{nW}}}/\sqrt{{{\text{Hz}}}}$, bit depth (ADC resolution) of 8 bits and the frame rate on the detector array can be set between 0.23 and 103 fps with corresponding exposure times. The bit depth introduces a theoretical digitization error of $\sim \, 0.4$% (i.e. − 27 dB) from the full range, hence it is important to adjust the exposure time to the incident power levels. During experiments we noticed that the acquired data in the video mode format are sampled with only 6 effective bits, introducing additional quantization error. Details can be found in “Accuracy considerations” in “Discussion” section.

The variable phase delay is introduced into the reference arm via a corner reflector on an electronic linear translation stage. The shift of the mirror $\Delta L$ results in a phase shift of $\phi _{{\text{R}}} = 2 k \Delta L$, where $k = 2\pi /\lambda$ is the wavenumber and $\lambda =1.03~\hbox {mm}$ is the wavelength in free space. In the experiment, the stage moves with speed of 0.5 mm/s by $\Delta L = 0.5\,\hbox {mm}$ giving a maximum phase shift of a little below $2\pi$. As the translation stage moves, we record the signal with the detector array with a frame rate of 25 frames/s.

After the 1 s data acquisition time, a one-dimensional Fourier transform is evaluated from the time-domain signal sampled at each individual pixel and the measured phase $\phi _{{\text{M}}}(x,y) = \phi _{{\text{R}}}(x,y) - \phi _{{\text{A}}}(x,y)$ of the Fourier component on the desired position in the spectrum is read out. The expected oscillation frequency of this component can be pre-determined as $f_{\Delta L}=1 / \lambda$. The measured phase relates the phase of the unknown beam $\phi _{{\text{A}}}$ to the phase distribution of the known reference field $\phi _{{\text{R}}}$ in the plane of the detector. If $\phi _{{\text{R}}}$ is known $\phi _{{\text{A}}}$ can be evaluated. In our measurements we approximate the reference field by a spherical wavefront with origin located in the aperture of the horn antenna and with radius of curvature 1.56 m (see Fig. 3c), corresponding to the path length between the horn’s aperture and the centre of the detector array following the reference path in Fig. 2.

The normalized amplitude $E_{{\text{A}}}$ is also determined from the amplitude of the spectral component $A_{{{\text{M}}}}(x,y)= 2|E_{{\text{R}}}(x,y)| |E_{{\text{A}}}(x,y)|$ (i.e. amplitude of the last term in Eq. 1) as the reference amplitude $|E_{{\text{R}}}|$ can be determined by blocking the AUT beam, leaving only the reference beam incident onto the detector. If stable, the non-uniform and distorted amplitude distribution of the reference field is not decisive for the accuracy of the measured unknown amplitude $E_{{\text{A}}}$. However, an accuracy issue arises when the amplitude of the reference field changes with the delay line position $\Delta L$, e.g. as in our case due to combination of non-perfect mirrors, small optical misalignment and reference beam’s divergence and interference effects due to stray radiation.

Alternatively, the unknown amplitude can be measured directly by blocking the reference path. It can be calculated from the measured intensity $|E_{{\text{A}}}(x,y)| = \sqrt{I_{{\text{A}}}(x,y)}$ from a single frame recording. Since the responsivity of the individual pixels is not identical (see Fig. 3a) the measured amplitude can be normalized by a measured responsivity map, obtained from uniformly illuminated detector array (see Fig. 3a). We do not attempt this as the data in Fig. 3 has been acquired and averaged over much longer timescale compared with the experiment and thus serve for illustration only.

**Fig. 4:**
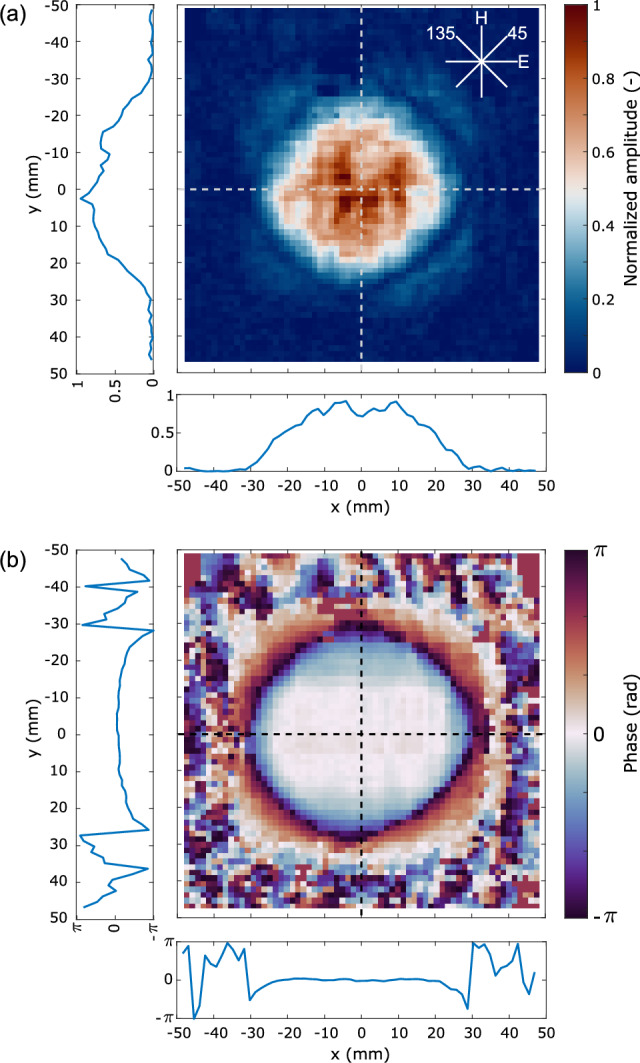
Measured E-field distribution of the unknown beam. (**a**) Normalized, measured amplitude distribution $|E_{{\text{A}}}(x,y)|$ including the 1D cuts on the bottom and left-hand side. (**b**) Measured phase distribution $\phi _{{\text{A}}}(x,y)$ including the cuts.

The two-dimensional amplitude $|E_{{\text{A}}}(x,y)|$ and phase $\phi _{{\text{A}}}(x,y)$ distributions of the AUT can be found in Fig. 4a,b, respectively. The one-dimensional cuts through the centre of the beam (along the dashed lines) are displayed on the left and on the bottom of the 2D plots. The ripple in the amplitude was influenced by the mechanical properties of the delay line mirrors (e.g. planarity) and possible scattering withing the cover layers of the camera pixels. Further contributing factors were identified as the imperfect calibration caused by pixels’ non-uniform responsivity as well as partial reflections in the optical setup—i.e. the detector array reflects back about 10% of the incident power. However, the main factor contributing to the patchiness/pixelization in the plots is the limited dynamic range given by the low bit depth of the pixels (for details see “Accuracy considerations” in “Discussion” section). The phase distribution shows uniform phase across the horizontal cut (H-plane) and a slight curvature across the vertical direction (E-plane). A small asymmetry in the curvature can be observed caused by a linear phase gradient superimposed onto the phase in this plane and given probably by a transversal misalignment of the feed with respect to the optical axis of the lens. The curvature in this plane could be either caused by the non-collocated E- and H-plane phase centres of the diagonal horn, or more likely by a curvature of the corner reflector on the delay line stage.

Extracting the complex fields from the $64 \times 64 \times 25$ dataset ($64 \times 64$ pixels and 25 time samples per pixel) took less than 2 ms on a modern desktop PC, representing only a negligible overhead.

### Far-field radiation pattern estimation

**Fig. 5:**
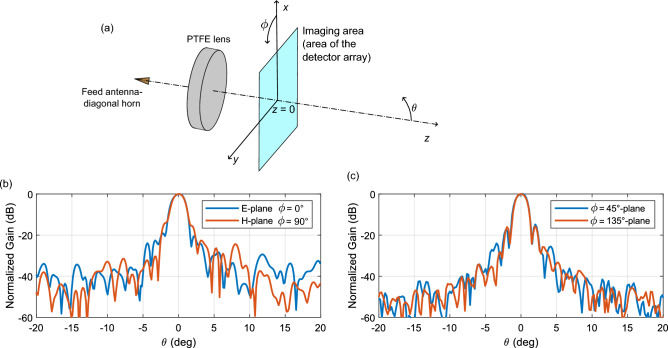
Estimation of far-field radiation patterns. (**a**) Spherical coordinate system for determining the radiation pattern of the AUT. (**b**) Normalized gain in the principal planes of the antenna (see Fig. 4). (**c**) Normalized gain on the diagonal planes.

A two-dimensional Fourier transform of the complex electric field distribution $E_{{\text{A}}}$ given in Fig. 4a,b is evaluated and reveals the plane wave spectrum of the antenna $A(k_x,k_y)$ (see Eq. (2)) as a function of in-plane wavenumbers, i.e. ${A(k_x,k_y) = {\mathcal {F}}\{E_{{\text{A}}}(x,y,z=0)\}}$, where $z=0$ corresponds to the axial location of the detector plane and ${\mathcal {F}}$ represents a 2D Fourier transform. We note that generally, the E-field and plane wave spectrum components are vector quantities, but here we only focus on the co-polarized component of the radiation of the antenna.

2 $$\begin{aligned} A(k_x, k_y) = \frac{1}{2\pi } \int \limits _{x_{min}}^{x_{max}} \int \limits _{y_{min}}^{y_{max}} E_{{\text{A}}}(x,y,z=0) {{\text{e}}}^{-i(k_xx+k_yy)} \,dx\,dy, \end{aligned}$$

where $x_{min}, x_{max}, y_{min}, y_{max}$ represent the vertical and horizontal boundaries of the area over which we image the fields—see Fig. 5a.

We can calculate the estimated far-field radiation pattern $F(\theta , \varphi )$ in a spherical coordinate system shown in Fig. 5a from the plane wave spectrum according to Eq. (3) (see Ref.^28^). These can be represented by normalized gain defined as $20 {{\text{log}}}_{10} \{F(\theta , \varphi )/{{\text{max}}}[F(\theta , \varphi )]\}$. For brevity, in Fig. 5a we include two orthogonal far-field cuts of normalized gain on the E- and H-planes corresponding to the vertical and horizontal cuts in Fig. 4b. The diagonal cuts at $45^\circ$ and $135^\circ$ planes are then given in Fig. 5c.

3 $$\begin{aligned} E_{ff}(r, \theta , \phi ) = {{\text{i}}} \frac{{{\text{e}}}^{-{{\text{i}}}k_0\,r}}{r}\,k_z\,A(k_x, k_y) = F(\theta ,\phi ) \frac{{{\text{e}}}^{-{{\text{i}}}k_0\,r}}{r}, \end{aligned}$$

where $E_{ff}$ is the electric field in the far field and the wavenumber components are related to the angles $\theta , \phi$ of the spherical coordinate system as

4 $$\begin{aligned} \begin{aligned} k_x&= k_0\,\sin {\theta }\,\cos {\phi },\\ k_y&= k_0\,\sin {\theta }\,\sin {\phi },\\ k_z&= k_0\,\cos {\theta }, \end{aligned} \end{aligned}$$

with $$k^2_0 = \sqrt{k^2_x+k^2_y+k^2_z}$$.

We note that prior to the Fourier transformation, the data is zero padded to size of $256\times 256$ from the original $64 \times 64$ to increase the smoothness of the plots. We observe that the beamwidth in the E-plane is slightly wider which is consistent with the curved phase profile in the vertical cut of Fig. 4b as well as with the beamwidth of the feed horn antenna.

## Discussion

### On the beam alignment and the spherical wavefront assumptions

**Fig. 6:**
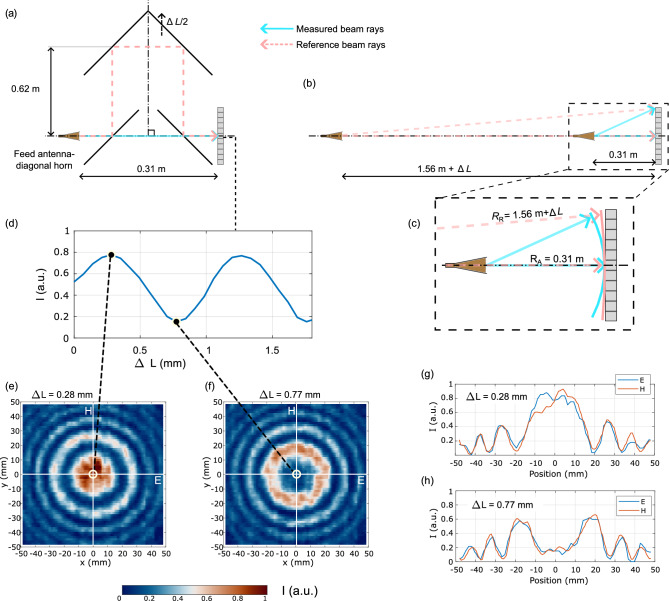
Alignment of the optics and the spherical wavefronts approximation. **(a)** The experimental setup with the PTFE lens removed. **(b)** The schematic of the experimental setup with the reference beam unfolded onto a line. **(c)** Radii of curvature of the two spherical-like wavefronts corresponding to the reference beam and measured beam. **(d)** Measured, normalized intensity profile at the centre of the camera with optical path length of the reference beam increasing by $\Delta L$. Measured interferograms at two positions of $\Delta L$ corresponding to maximum intensity at the central pixel **(e)** and minimum intensity at the central pixel **(f)** and the corresponding 1D cuts in **(g)** and **(h)**.

The optical axis of the measured AUT beam and of the reference beam are set up following Fig. 6a with the PTFE lens removed from the experiment. We rely on the interference of two spherical-like wavefronts generated by the feed antenna that follow two separate paths—either along the measured beam or along the reference beam (see Fig. 6a). In this configuration, the array detector is in the far field of the feed antenna in both paths, so the spherical approximation is reasonable. Figure 6b introduces an unfolded setup which is equivalent to the actual one from Fig. 6a, the reference beam’s axis is aligned with the measured beam axis and the optical path lengths (OPLs) are preserved. The equivalent setup simplifies the description of the alignment procedures. The camera records an interference pattern of two beams with spherical wavefronts but with different radii of curvature (Fig. 6c)—0.31 m for the measured beam and 1.56 m$+\Delta L$ for the reference beam, where $\Delta L$ corresponds to the increase in OPL as the corner reflector moves by the distance $\Delta L/2$. The distances in this case correspond to the rays connecting the interfering sources and the pixel in the centre of the FoV. The measured intensity at this pixel as a function of the increasing $\Delta L$ is plotted in Fig. 6d. In this case a different exposure setting was used with 14 fps refresh rate compared to the AUT measurement performed with 25 fps. We align the beams observing the symmetry and power distribution inside of the fringes of the interference pattern as we shift the corner reflector.

Figure 6e,f plot the imaged intensity at two different positions of the corner reflector resulting in constructive (e) or destructive (f) interference at the central pixel. We observe a circular symmetry in the fringes. A better quantitative comparison can be drawn from Fig. 6g,h where cuts across the two orthogonal planes are given for the two cases of constructive/destructive interference at the centre. Very good alignment of the peak maxima can be observed. We believe this also validates our assumption of spherical wavefronts of the reference beam to a degree sufficient for the demonstration purposes considered in this paper. Nevertheless, we do observe a slightly different positions of the outermost fringes in the E and H planes, which can be explained by the properties of the feed horn antenna. The estimate of the systematic phase error associated with this shift is $\lambda /10$ (see Fig. 6h, at positions about $\pm 45~\hbox {mm}$).

The alignment procedure is concluded when the centre of the interference fringes is in the centre of the camera and stays stationary with changing $\Delta L$, and the power is distributed symmetrically inside of the fringes along horizontal/vertical planes and stays stationary with changing $\Delta L$. Once the alignment is finished, we introduce the PTFE lens into the setup at position according to the Fig. 2b, so that the horn’s aperture is located in the back focal plane of the lens (back focal length of the lens $\approx 130~\hbox {mm}$).

### Accuracy considerations

Here, we estimate the cumulative effects of the imperfections and limitations of our setup on the accuracy of the measured data, via evaluating the spatially varying relative error in measured intensity *I*. Any multipath interference from stray radiation in the setup is also accounted for in this analysis. This originates from the non-perfect absorption of the microwave absorbers around the setup, from scattering/diffraction of the beam from the edges of components such as beamsplitters, as well as the partial reflection from the detector array itself, which we measured to be about 10% at normal incidence. In addition, detector noise is also accounted for in this analysis.

**Fig. 7:**
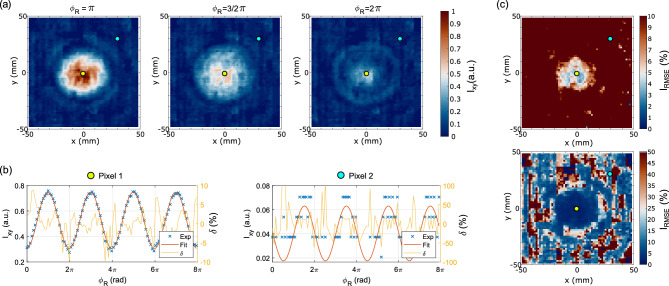
Accuracy of the measured intensity interferograms. (**a**) Three interferograms corresponding to reference beam phase shifts of $\pi$, $3/2\pi$ and $2\pi$. Two pixels are highlighted in these plots. (**b**) Harmonic oscillation of the intensity *I* in the central pixel (32,32) as a function of reference phase over an extended $8\,\pi$ range. Experimental and fitted data are compared and a relative difference is plotted as a function of phase shift. (**c**) Measured and fitted intensity at pixel 2 (51,51) showing an increased in relative error due to the quantization error.

Figure 7a shows three interferograms produced by the interference between the measured AUT beam and the reference beam in the configuration according to Fig. 2a that correspond to three distinctive phase shifts of the reference beam, i.e. $\pi$, $3/2\pi$ and $2\pi$. In Fig. 7b we plot the intensity values at a single pixel as the reference phase shifts by small steps ($\Delta \phi _R = 0.07\,\pi$), so that we can trace the accuracy as the reference phase changes, i.e. the translation stage moves by 2.1 mm. We examine this waveform at two locations within the field of view denoted as “Pixel 1”, located in the centre of the array and “Pixel 2”, located in the upper right corner of the array. The effects of the limited dynamic range set by the maximum intensity and resulting quantization error at pixels with much smaller intensities becomes obvious—see Fig. 7b. The measured data points at Pixel 2 also show that the quantization error is much larger than anticipated, we only seem to be resolving 64 values instead of 256. We note that this probably occurs because of sub-optimal camera settings related to the colorscale used during the acquisition and is potentially the most straightforward way to increase the accuracy of the measurement.

The relative errors $\delta$ in Fig. 7b are calculated as a difference between the measured data point $I_{{\text{M}}}(x,y,\phi _R)$ and a fitted value $I_{{{\text{Fit}}}}(x,y,\phi _R)$ normalized to the fitted intensity value:

5 $$\begin{aligned} \delta (x,y, \phi _R)= \frac{I_{{\text{M}}}(x,y,\phi _R) - I_{{{\text{Fit}}}}(x,y,\phi _R)}{I_{{{\text{Fit}}}}(x,y,\phi _R)}, \end{aligned}$$

and in order to evaluate the approximate error over the mechanical shift of the corner reflector, i.e. over the $8\,\pi$ phase shift of the reference beam we calculate the error of the *I* value in a root-mean-square fashion:

6 $$\begin{aligned} I(x,y)= \sqrt{\frac{1}{n} \sum _{\phi _R = 0}^{8\pi } \delta (x,y,\phi _R) }, \end{aligned}$$

where $n=102$ is the number of the measured data points in Fig. 7b. Figure 7c plots this error as a spatially varying quantity with either 10% saturation value or 50%. We can see how the areas with the smallest intensity values show the highest levels of error. Reduction of the quantization error of the ADC is the most simply rectifiable limitation of our setup which could reduce the error fourfold and should be achievable under optimized settings of the camera used in the experiments.The limited dynamic range of the acquisition (6-bit depth during our measurements) can be improved by using techniques such as multi-exposure high dynamic range capture^29^, where multiple exposures of the same scene are combined at the expense of increased acquisition time.

We also see some evidence of minor interference artifacts from imperfect lenses and reflectors in our setup. Large aberration free collimation optics are necessary to provide necessary intensity in the camera plane at given exposure setting, while calibration of the camera under various angles of incidence would normalise angular sensitivity. Similarly, a non-mechanical phase shifter, e.g. a transmission line based solution or a modulation of refractive index of bulk material (e.g. silicon or liquid crystals), would also reduce interference artifacts which change when physically moving the reflector. Finally, while the diameter of the Si beamsplitter close to the feed horn antenna source is sufficient as the beam is relatively small at this position, the size of the beam combiner, i.e. the Si wafer closer to the camera in Fig. 2a is not large enough and we can see it clips the reference beam at the edges—corners in Fig. 3b, causing insensitivity of these pixels and introduces diffracted fields into the FoV. In order to minimise artifacts, a larger diameter beam combiner close to the camera to avoid beam clipping and diffraction.

A final interesting consideration is the reference antenna. A well characterized reference source in terms of amplitude and phase distribution is necessary for any metrology applications. We used a diagonal horn antenna with unknown position of the phase centre in the experiment. The phase distribution was thus assumed to be perfect spherical with origin in the aperture of the horn. From the measured data, we can see this is not exactly true. For example, in the interference plots in Fig. 6g,h we can see the interference maxima in the outermost regions on the two principal planes do not perfectly coincide. We can trace this to either the non-collocated phase centre in the E and H planes of the source or to a slightly curved corner reflector of the delay line stage. A better characterized reference antenna would help improve the accuracy of the results.

### Comparison with probe-scanning techniques

The standard technique for planar near-field antenna characterization, mentioned earlier in the paper, relies on a single probe antenna scanning the area close to the antenna using an *xy* translation thus mapping the electric field distribution at certain *z* coordinate $E_A(x, y, z)$. At each transverse location (*x*, *y*), the probe stops and integrates the signal over some chosen time. A plane wave spectrum is then calculated and from it a far-field radiation patterns are derived, as explained in the section above.

The time required to run the scan is dominantly given by the probe speed, scanning area and time constant of the VNA and usually is not listed in research publications. As such a direct comparison is very difficult. To better illustrate the potential measurement speed-up with our technique we can mention two systems operating in similar frequency range as ours^30,31^. In Ref.^30^ authors only list the scanning speed of 6 mm/s to map the near field across $16 \times 16\,\hbox {mm}^2$ at 220–325 GHz, but they do not mention the type and specifics of acquisition. The equivalent measurement time for the area corresponding to the one this paper $96 \times 96\,\hbox {mm}^2$, corresponds to 1024 s (16 s per row, 64 rows). In Ref.^31^ the authors implement an on-the-fly version of scanning, where the probe continuously scans the area instead of parking at each position. The authors present a solution $2.5\times$ faster compared to standard implementation and they scan near-fields of a 500 GHz antenna over area of $80 \times 80\,\hbox {mm}^2$ in 900 s.

There have been multiple concepts proposed to speed up the field scanning process as outlined in Ref.^32^. These rely on sparsing the number of measurement positions through e.g. compressed sampling^33,34^, reducing the scan area and most importantly on multi-probe approach where multiple probes are used to increase the scanning speed. Such a multiprobe concept is conceptually developed in Ref.^32^ for Ku band antennas using 9 probes distributed in $3 \times 3$ array and scanned on a spiral path to sample the required area thus potentially speeding the data acquisition by a factor of 9. However, as pointed out by the authors, a multi-channel VNA is necessary for the implementation of the technique.

Considering our approach, we can see that the technique described in this paper completely removes the need for mechanical scanning of the detector and thus has the potential to become more robust. The one-second acquisition time obtained in our experiment is about three orders of magnitude below the standard techniques^30,31^ under comparable conditions and about two orders of magnitude if we considered augmentation with a multi-probe approach^32^.

It could be considered that our technique extends the number of the probes from 9 as used in Ref.^32^ to 4096. However, an important difference in our work is the use of holographic detection with amplitude only detectors, which removes the stringent requirement on the coherent detectors required by multi-probe approach.

## Conclusion

We have demonstrated how phase-shifting digital holography can be implemented to allow for real-time beam profiling of complex fields and rapid near-field antenna characterization. An off-the-shelf detector array was used to parallelize the data acquisition over the detection plane (96 $\times$ 96 $\hbox {mm}^2$) together with free-space distribution of the reference field. As the post-processing of the measured data via 1D Fourier transform takes only a few ms, the imaging speed was dictated by the 1 s acquisition time, during which the delay line stage moved by 0.5 mm, resulting in frame rate of about 1 Hz, compared to tens of minutes required for typical probe scanning technique. We note that the speed can be substantially improved as the translation stage moved at a low speed in our experiment so that the acceleration/deceleration had negligible effects on the measurement.

Using this technique, we measured the amplitude and phase of a beam radiated by a diagonal horn-fed spherical lens antenna in its radiative near field. The far-field range defined by $2D^2/\lambda$, where $D=100\,\hbox {mm}$ is the diameter of the lens starts at about 20 m away from the antenna. The measurement was conducted at the frequency of 290 GHz with 1 Hz frame rate and we estimated the normalized far-field radiation patterns via 2D Fourier transform of the measured beam. This technique can be extended to absolute gain measurements once a well-characterized source and reference antenna are used in a two or three antenna gain measuring method^35^. Further investigation into the polarization purity of the individual pixels will be required for possible cross-polarization measurements. The limited dynamic range of the acquisition ($\sim$ 21 dB) can be improved by using techniques such as multi-exposure high dynamic range capture^29^, where multiple exposures of the same scene are combined at the expense of increased acquisition time.

We believe that despite the limited accuracy, arising from the pixel response of the detector array and the mechanically imperfect reference, this technique has a potential to serve as a rapid and compact characterization tool in estimating the performance of terahertz antennas and quasi-optical systems and thus complement highly accurate, but slow, VNA-based, probe-scanning techniques.

## Data Availability

The datasets used and/or analysed during the current study available from the corresponding author on reasonable request.
